# Vitamin B12 Status and Gut Microbiota among Saudi Females with Obesity

**DOI:** 10.3390/foods11244007

**Published:** 2022-12-11

**Authors:** Sara Al-Musharaf, Ghadeer S. Aljuraiban, Lama Al-Ajllan, Noura Al-Khaldi, Esra’a A. Aljazairy, Syed Danish Hussain, Abdullah M. Alnaami, Shaun Sabico, Nasser Al-Daghri

**Affiliations:** 1 Department of Community Health Sciences, College of Applied Medical Sciences, King Saud University, Riyadh 11451, Saudi Arabia; 2 Chair for Biomarkers of Chronic Diseases, Riyadh Biochemistry Department, College of Science, King Saud University, Riyadh 11451, Saudi Arabia

**Keywords:** serum vitamin B12, dietary vitamin B12, cobalamin, gut microbiota, microbiome, obesity

## Abstract

Previous studies have suggested that dietary habits and dysbiosis of gut microbiota contributed to obesity development. Vitamin B12 is produced by microbes; however, the relationships between vitamin B12, gut microbiome, and obesity are understudied. We aimed to determine the association between vitamin B12 status and gut microbiota relative to obesity in 92 Saudi Arabian females aged 19–25 years who were obese (n = 44) or normal weight (n = 48). Anthropometric, biochemical data, and dietary data were collected. The microbial communities of stool samples were characterized using the shotgun metagenomic sequencing technique. The relationship between vitamin B12 status and gut microbiota composition was identified using Pearson correlation analysis. A statistically significant difference was found in bacterial α- and β-diversity between the groups relative to median serum vitamin B12 level (404.0 pg/mL) and body weight. In the total participants, dietary vitamin B12 intake was inversely correlated with *Bifidobacterium kashiwanohense* and *Blautia wexlerae* species. In obese participants, dietary vitamin B12 intake was inversely correlated with *Akkermansia muciniphila* species and species from the Verrucomicrobia phylum, whereas it was positively correlated with Bacteroides species. Our findings indicate that the abundance (frequency) and diversity (richness) of gut microbiota are associated with vitamin B12 levels and obesity in young females.

## 1. Introduction

Obesity is a major worldwide public health concern [1]. In 2016, 13% of the adult global population was obese and 39% were overweight [1]. According to the 2019 Kingdom of Saudi Arabia World Health Survey, the prevalence of obesity and overweight in Saudi Arabia reached 20% and 38%, respectively, with more females than males being obese (21% vs. 19%) [2]. The increasing prevalence of overweight and obesity is a concerning public health issue especially for women [2], and has been related to infertility [3] and the occurrence of chronic diseases in both mothers and their offspring [4]. Several other health problems are also associated with obesity, such as, cancer, cardiovascular diseases, and metabolic syndrome [1]. Obesity is considered a multifactorial disease, with sedentary lifestyle and disturbed macronutrient intake among the most prominent contributing factors [1]. 

Recently, other risk factors have gained attention, including micronutrient deficiencies and altered gut microbiota [5,6]. Microbiota, a collection of microorganisms that constitute a particular ecological community [7], are mainly affected by diet, which can impact gut diversity and composition [8,9]. While considerable attention has been given to the macronutrient–gut microbiota relationship [9], studies have rarely focused on the role of vitamins (e.g., vitamin B12) [10,11]. Vitamin B12 insufficiency (<200 pmol/L) is a common condition worldwide [12], reaching an incidence of 10.6% in the United States [13] and 6% in Saudi females of reproductive age (≤220 pmol/L) [14]. Vitamin B12 insufficiency has been linked to a variety of health problems, ranging from minor tiredness and megaloblastic anemia to severe neurological damage [12], as well as the development of metabolic diseases, particularly obesity [15]. Previous studies have suggested that vitamin B12 insufficiency was associated with abnormal lipid profiles [16] and obesity [17]. Insufficiency of vitamin B12 may be due to low dietary intake and malabsorption [18], in addition to altered gut microbiota, since vitamin B12 acts as a metabolic cofactor for gut microbes and is exclusively synthesized by bacteria [19]. Interestingly, the association between vitamin B12 levels and gut microbiota may be bidirectional [19]. The gut microbiota community balance and functional interaction may be disrupted due to the lack of vitamin B12 [20]. Consequently, vitamin B12–gut microbiota interactions may prevent the development of obesity by altering the microbial composition of the gut [21]. Therefore, vitamin B12 status, gut microbiota, and obesity may be interrelated.

Available studies have focused exclusively on single relationships between obesity and gut microbiota [22], obesity and vitamin B12 [17], and microbiota and vitamin B12 [19]. To our knowledge, limited studies have assessed the combined relationships between serum vitamin B12, gut microbiota, and obesity. With the current shift in the diet of the Saudi Arabian population from traditional to Western cuisines [23], and the rising obesity epidemic, a better understanding of the relationships between nutrient levels, gut microbiota, and adiposity is important. The present study aimed to identify associations between vitamin B12 status and the gut microbiota in Saudi Arabian females with regard to obesity levels using the shotgun metagenomic sequencing technique to measure gut microbiota composition and diversity 

## 2. Materials and Methods

### 2.1. Study Design

The current analysis is part of an assessment of gut microbiota and adiposity conducted between January 2019 and March 2020 [24]. In the current analysis, we investigated dietary data, particularly vitamin levels, in relation to microbiota and obesity. Details on study methodology has been published [24]. In brief, multimedia channels were used to recruit participants aged 18 years and above who are free from chronic diseases. The exclusion criteria were as follows: age ≤ 18 years old, pregnancy, gastrointestinal diseases (such as inflammatory bowel disease and acute or chronic diarrhea), history of oncological or endocrine disease, psychiatric disorders, other medical conditions, and use of vitamin supplements or antibiotics in the six months preceding stool sampling. We excluded n = 308 participants for several reasons, including refusing to provide stool samples, not reverting the stool container, or having DNA concentration below the required level. The total sample population who met the criteria (n = 92) were aged 19 to 25 years and were divided into two strata: (n = 44, Body Mass Index (BMI) ≥30 kg/m^2^) and (n = 48, 18.5–24.9 kg/m^2^). All recruited participants signed a consent form before being included in the study. After that, participants were provided with a container for stool samples, which was returned the same day the sample was taken. Each participant was asked to book an appointment at the nutrition clinic to complete a comprehensive assessment, including anthropometric measurements, dietary data, bioelectrical impedance analysis, blood sample collection, and study questionnaires. The study was carried out at King Saud University (KSU) after being approved by the KSU institutional review board (IRB #E-19-3625).

### 2.2. Data Collection

Each participant booked an appointment at the nutrition clinic to perform a full assessment, which took approximately 45 min. A trained dietician conducted an interview to collect relevant sociodemographic, lifestyle factors, and dietary data. Finally, participants were provided with collection containers for stool samples, which were returned the same day the sample was taken.

#### 2.2.1. Anthropometric Measurements

Trained clinical dietitians collected all anthropometric measurements in duplicate using standardized methods, and the average figure was used in the analysis. Weight and height were measured using an international scale (Digital Pearson Scale; ADAM Equipment Inc., Oxford, CT, USA). The calculation of the BMI was obtained by dividing weight in kilograms by height in meters squared. Participants were classified into two groups: normal weight (18.5–24.9 kg/m^2^) and obese (≥30 kg/m^2^) based on the WHO criteria (2018). Non-stretchable tape was used to measure waist and hip circumference, with the midway between the lowest rib and the umbilicus for waist circumference, and the great trochanter with the legs close together for hip circumference. In addition, the waist–hip ratio (WHR) was calculated by dividing the mean waist circumference by the mean hip circumference [25]. Body fat percentage (BF%) and muscle mass were measured via bioelectrical impedance analysis (BIA; 70 Bioelectrical Impedance Analyzer; InBody, Seoul, Republic of Korea) [26]. 

#### 2.2.2. Biochemical Measurements

Blood samples were collected after an overnight fast (≥10 h) to measure serum levels of vitamin B12, fasting blood glucose (FBG), insulin, and lipid profile, including high-density lipoprotein cholesterol (HDL-C), total cholesterol (TC), and triglyceride (TG) levels. Samples were transported to the study lab. A biochemical analyzer was used to determine the lipid profile (Konelab, Espoo, Finland). Low-density lipoprotein cholesterol (LDL-C) was computed based on a previously published formulae [27]. A LIAISON XL analyzer was used to evaluate insulin levels (DiaSorin, Saluggia, Italy). The homeostatic model assessment of insulin resistance (HOMA-IR) index was then computed [28]. Serum vitamin B12 levels were measured using electrochemiluminescent immunoassays using a Roche Cobas e411 immunoassay analyzer (Roche Diagnostics, Mannheim, Germany). 

#### 2.2.3. Stool Analysis 

Under sterile conditions, stool samples were collected in a clean container with dry screw-top and transported to the study lab. 

##### DNA Extraction

The DNA was extracted from 1.5–2.0 frozen stool aliquots using the QIAGEN PowerFecal DNA Kit (Catalogue: 12830-50). The DNA was eluted in 100 microliters of the C6 elution buffer according to the kit’s protocol. Using a NanoDrop spectrophotometer, the isolated DNA purity (260/280a ratio) and concentration (≥1.6) were measured (Thermo Fisher Scientific, Waltham, MA, USA).

The extracted DNA samples were preserved at −20 °C then transported in a package with dry ice to the CosmosID company located in the USA (CosmosID Inc., Rockville, MD, USA) for library preparation, sequencing, and bacterial composition identification.

##### Library Preparation and Sequencing

DNA libraries were constructed using the Nextera XT DNA Library Preparation Kit (Illumina, San Diego, CA, USA) and Nextera Index Kit (Illumina) with total DNA input of 1 nanogram. A proportional amount of Illumina Nextera XT fragmentation enzyme was used to fragment genomic DNA. Combinatory dual indexes were provided to each sample, followed by 12 cycles of each sample, which provided a combination of dual indexes, followed by 12 cycles of polymerase chain reaction (PCR) to build libraries. AMpure magnetic Beads (Beckman Coulter, Brea, CA, USA) were used to purify DNA libraries, which were then eluted in QIAGEN EB buffer. For the quantitative evaluation, a Qubit ^®^ fluorimeter (Thermo Fisher Scientific, Milan, Italy) was used. Following the library preparation, samples were sequenced on an Illumina HiSeq 4000 (2 × 150 bp). 

##### Identification of the Microbial Composition

The gut microbiota composition was determined at the level of the major microbial phyla and was carried out by identifying the total bacterial DNA and the Bacteroidetes and Firmicutes DNA using shotgun metagenomic sequencing [29], and was then analyzed using CosmosID bioinformatic platform (CosmosID Inc., Rockville, MD, USA). Shotgun metagenomic sequencing is considered a gold-standard technique for assessing the composition and diversity of the gut microbiota [29]. 

#### 2.2.4. Dietary Intake

Macronutrients and micronutrient consumption for the past year were assessed for each participant using the Saudi Food and Drug Authority’s food frequency questionnaire (SFDA-FFQ) [30]. The questionnaire was developed in the Arabic language and used a closed-ended approach, including a list of 133 food items [30]. The SFDA-FFQ was analyzed using Food Processor Nutrition Analysis Software version 11.1 (ESHA Research, Salem, OR, USA). Additionally, the researchers used an additional questionnaire that was developed specifically to estimate vitamin B12 levels from food and beverages [31]. The recommended dietary allowance (RDA) of vitamin B12 for adults is 2.4 mcg/day and the estimated average requirement (EAR) of vitamin B12 for adults is 2.0 mcg/day [18].

### 2.3. Statistical Analysis

The statistical analysis was done using IBM SPSS Statistics for Windows (version 24; IBM Corp., Armonk, NY, USA). Participants were divided into two groups using the median of serum vitamin B12 level [404.0 (311.0–632.2) pg/mL]. The intra- and inter-assay coefficient of variation values were 2.9% and 4.1%, respectively. Furthermore, participants were divided into four subgroups based on BMI and vitamin B12 serum. The normality of each quantitative variable was tested before the analysis. Appropriate non-parametric tests were used if variables showed a skewed pattern. Descriptive analysis results are presented as percentages for categorical data and means and standard deviations for continuous data. The independent samples t-test was used to assess the differences between continuous variables, while chi-square analysis was used to compare categorical variables. Dietary data are presented in g/1000 kcal or g/day.

Kruskal–Wallis test (one-way analysis of variance) was used to determine the differences between subgroups and compare median abundances of each phylum as it presents the percentages of the total microbial sequences. To further understand the relationship between vitamin B12 status and gut microbiota composition, the Pearson correlation analysis was applied. To describe the richness and evenness of microbiome, Shannon–Wiener index was used to compare bacterial α-diversity between the microbiota of normal-weight and obese participants based on BMI and serum vitamin B12 status. To evaluate and visualize the β-diversity, which measures similarity or dissimilarity between communities, principal coordinate analysis (PCoA) plots were constructed using the Bray–Curtis index. A *p*-value of <0.05 and a 95% CI were used to report the statistical significance and precision of the estimates. For missing laboratory values, we used mean imputation method to obtain the missing data [32].

## 3. Results

### 3.1. Anthropometric and Biochemical Analysis

A total of 92 females (mean age 21.1 (SD ± 1.5) y) were included in the analysis. General characteristics, including anthropometric, biochemical, and dietary data, are presented in Table 1 stratified by serum vitamin B12 status. Anthropometric data, including weight, BMI, and WHR, were higher in participants with low serum vitamin B12 levels (≤404.0 pg/mL) in comparison with those with a high serum level (>404.0 pg/mL) (*p* <0.05). Total cholesterol, LDL, triglycerides, insulin, and insulin resistance were significantly higher in participants with low serum vitamin B12 levels compared with those with serum levels higher than the median (Table 1). When participants were stratified into four groups based on BMI and vitamin B12 serum, a higher muscle mass percentage was observed in the normal-weight group compared with the high vs. low serum vitamin B12 group (*p* = 0.03) (Table 1). Among participants with obesity, serum levels of total cholesterol, LDL cholesterol, and TG were significantly higher among participants with low serum vitamin B12 compared with high serum levels (*p* = 0.01, *p* = 0.02, *p* = 0.03, respectively). No differences were observed among normal-weight participants (Table 1).

### 3.2. Dietary Intake and Lifestyle Characteristics

Total fat intake was significantly lower in participants with high serum vitamin B12 compared with low serum vitamin B12 in the group with obesity (35% vs. 42%, *p* = 0.03) (Table 1).

When participants were categorized based on vitamin B12 serum level only, the total intake of fat was significantly higher in participants with low serum vitamin B12 levels compared with those with higher serum vitamin B12 levels (*p*= 0.04) (Supplementary Table S1). Participants with low serum vitamin B12 had lower intakes of fruit (79 vs. 127 gm/1000 kcal, *p* = 0.04) and chicken (56 vs. 88 gm/day, *p* = 0.02) in comparison with participants with high serum vitamin B12 levels. No significant differences were found for other dietary intakes between the groups (Supplementary Table S1).

### 3.3. Differences in Gut Microbiota Composition across Subgroups 

Fecal analyses of participants with low and high serum vitamin B12 levels relative to body weight are presented in Table 2. There were no statistically significant differences between the groups with regard to gut microbiota phylum and species. However, in normal-weight participants, there was a non-significant trend in higher Verrucomicrobia (*p* = 0.16) phylum in participants with high serum vitamin B12 compared with those with low serum vitamin B12. In normal-weight participants, a higher abundance of *Akkermansia muciniphila* (Verrucomicrobia phylum) was observed in participants with high serum vitamin B12 levels in comparison with low vitamin B12; however, it was not significant (*p* = 0.12). 

In obese participants, higher *Faecalibacterium prausnitzii* (Firmicute phylum) was also observed in participants with high serum vitamin B12 levels in comparison with participants with low serum vitamin B12 levels, although it was not statistically significant (*p* = 0.19) (Table 2).

#### 3.3.1. Correlation between Vitamin B12 (Serum and Dietary) and Gut Microbiota Composition

In the total participants, no significant correlations were found between gut microbiota and serum vitamin B12 (*p* > 0.05). 

**Figure 1:**
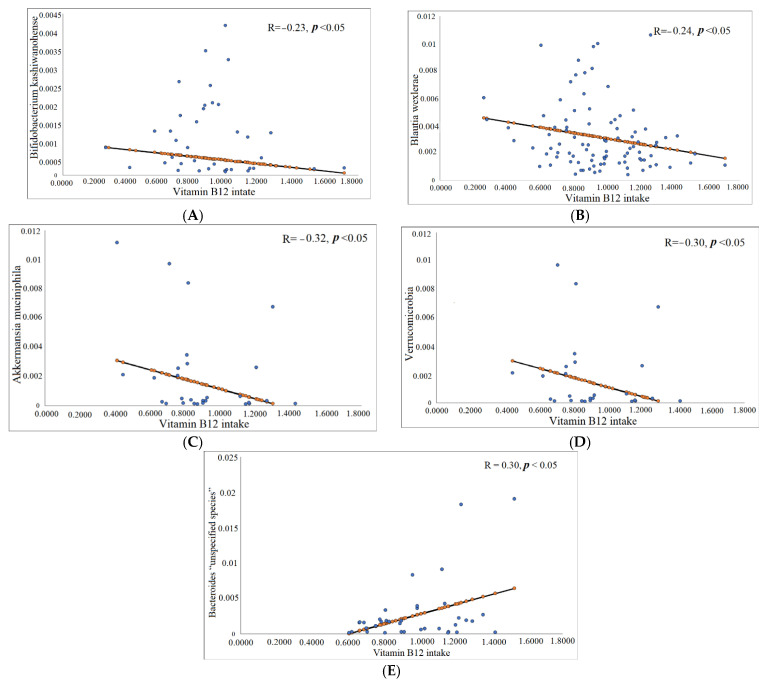
Correlation coefficients between vitamin B12 intake and (**A**) Bifidobacterium kashiwanohense species and (**B**) Blautia wexlerae in total participants. Correlation coefficients between vitamin B12 intake and (**C**) Akkermansia muciniphila species, (**D**) Verrucomicrobia phylum, and (**E**) Bacteroides “unspecified species” in obese participants (cases).

However, dietary vitamin B12 was inversely correlated with *Bifidobacterium kashiwanohense* species (Actinobacteria phylum) (r = −0.23, *p*= 0.03) and *Blautia wexlerae* (Firmicutes phylum) (r = −0.2, *p*= 0.02) in the total participants (Table 3, Figure 1A,B). In obese participants, an inverse correlation was observed between *Akkermansia muciniphila* species, Verrucomicrobia phylum, and dietary vitamin B12 intake (*r* = 0.32, *p* = 0.03 and *r* =0.30, *p* = 0.04) (Table 3, Figure 1C,D). Furthermore, there was a positive correlation between dietary vitamin B12 intake and *Bacteroides “unspecified species”* (*r* = 0.30, *p* = 0.04) (Table 3, Figure 1E).

#### 3.3.2. Abundance and Diversity

**Figure 2:**
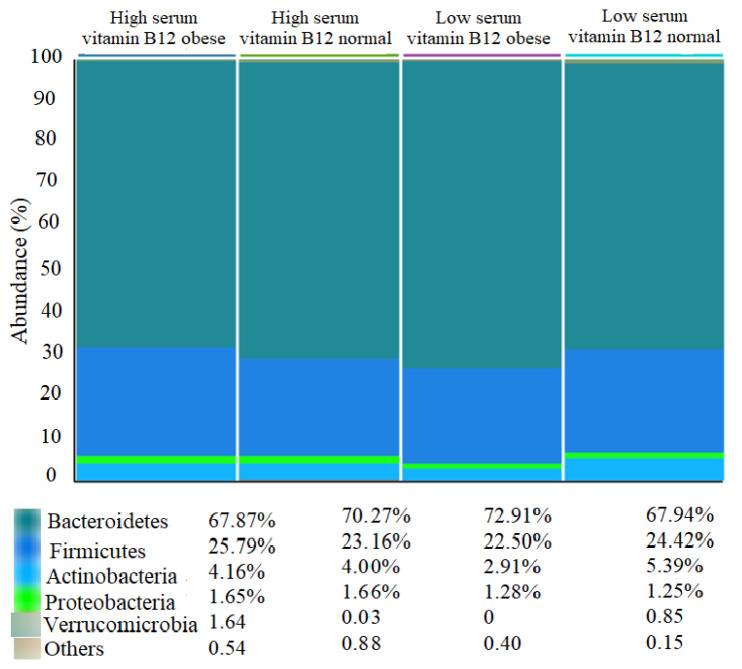
Classification of gut microbiota at phylum-level according to categories of serum vitamin B12 status relative to BMI.

The most abundant phyla in obese participants with low serum vitamin B12 were Bacteroidetes (72.91%), Firmicutes (22.5%), and Actinobacteria (2.91%) (Figure 2).

**Figure 3:**
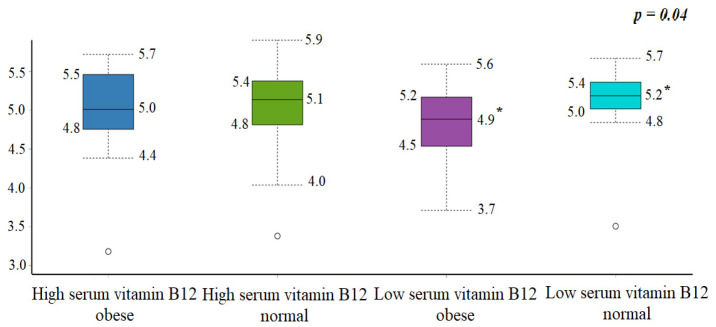
Comparison of bacterial α-diversity (Shannon–Wiener index) between the microbiota of normal-weight and obese participants based on BMI and serum vitamin B12 status (<404.0 pg/mL). * *p* < 0.05.

The Shannon–Wiener index showed significant differences in α-diversity between participants with high compared to low serum vitamin B12 levels in the obesity and normal-weight groups. Participants with low serum vitamin B12 levels in the obesity group had lower α-diversity than the normal-weight group (median: 4.9 vs. 5.2; *p* = 0.04, Figure 3). 

The principal coordinate analysis (PCoA) plots based on Bray–Curtis showed a significant difference in β-diversity shown by PC1, PC2 and PC3 accounting for 9.99%, 14.68% and 6.69% (Bray–Curtis index, *p* = 0.01). Result revealed a significant difference in β-diversity between high serum vitamin B12 in the normal-weight group compared to low serum vitamin B12 in the obese group.

## 4. Discussion

In this study, we explored the association between vitamin B12 status and gut microbiota among young Saudi females in association to body weight. We found a significant difference in both the α- and β-diversity of the gut microbiota in association with BMI and serum vitamin B12 levels. Additionally, we observed a non-significant trend of higher *Faecalibacterium prausnitzii* (Firmicute phylum) in obese participants with high serum vitamin B12 levels in comparison with participants with lower serum levels. In regard to dietary vitamin B12, we found that dietary vitamin B12 intake was significantly inversely correlated with *Akkermansia muciniphila* species and Verrucomicrobia phylum, and positively with Bacteroides “unspecified species” in participants with obesity.

Few studies have investigated α- and β-diversity in relation to vitamin B12 status in humans [33,34,35,36,37,38]. A systematic review by Guetterman et al. reported that serum vitamin B12, dietary intake, or supplementation may be associated with changes in bacterial abundance and may increase α-diversity [19]. However, studies on the association between α- and β-diversity and vitamin B12 status are limited: three were observational studies on humans [34,37,38] and the others were clinical trials on animals [34,35,36]. Notably, the majority only assessed vitamin B12 intake [33,34,35,36,37], with only two studies measuring serum vitamin B12 levels [37,39] and one other investigating the relation with α- and β-diversity [37]. However, several studies have explored α- and β-diversity with obesity internationally [40,41] and locally [42,43]. To our knowledge, no studies were found that link vitamin B12, gut microbiota, and weight.

This study reported that participants with low serum vitamin B12 in the obese group had significantly lower α- diversity than the normal-weight group. Low α-diversity is considered an accurate predictor of disease-associated dysbiosis [44]. Furthermore, there was a significant difference in β-diversity between high serum vitamin B12 in the normal-weight group compared with low serum vitamin B12 in the obese group. Β-diversity measures the composition that distinguishes microbial community between healthy and diseased participants [19], indicating the presence or absence of some uncommon species in the assemblages [19]. Up to date, there were two studies that assessed serum vitamin B12 and gut microbiota [37,39], and only one of them looked into α- and β-diversity [37]. Similar to our results, the first study was on infants, showing that the most abundant phyla in all participants were Bacteroidetes, Proteobacteria, Actinobacteria, and Firmicutes [37]. Contrary to our findings, they reported no differences in the microbiota α- and β-diversity between infants with vitamin B12 sufficiency or insufficiency, even after vitamin B12 supplementation [37]. The different results in bacterial diversity could be explained by the different age group, since in infancy vitamin B12 deficiency effects may not be fully established. Additionally, the second study was on serum vitamin B12 levels and probiotic supplementation, which reported significant increases in serum levels of vitamin B12 (*p* = 0.001), with an increase of bifidobacteria in participants who consumed an abacterial blend [39]. It is noteworthy that our study included obese and normal-weight groups. Hence, participants with obesity and low serum vitamin B12 had a specific gut microbiota profile compared with those with normal weight and high serum vitamin B12, suggesting an interplay in the role of gut microbiota and low serum vitamin B12 in obesity development among the Saudi population. 

Regarding vitamin B12 intake and α- and β-diversity, Kelly et al. conducted an experimental study of male and female mice that showed an inverse association between β-diversity and vitamin B12 supplementation (30.46 mcg/mouse) [35]. Additionally, a cross-sectional study among older male adults showed a significant difference in both the α- and β-diversity of gut microbiota in association with a higher dietary consumption of vitamin B12 (>2.38 mcg) among 35 participants, with 25 of them having obesity [38]. However, in China, a trial among a cohort of 773 overweight individuals and 997 normal-weight adults revealed that vitamin B supplementation had no effect on microbial α-diversity among the total sample [33]. However, the exacted form of vitamin B ingested by the participants was not specified and serum vitamin B12 was not measured [33]. Additionally, in an in vivo study of three-week-old mice that measured α-diversity using the Shannon index and β-diversity using Bray–Curtis dissimilarity and Binary Jaccard similarity [34], no significant difference in α- and β-diversity was found between the vitamin B12 groups after four weeks [34]. Nevertheless, the association between gut microbiota and vitamin B12 status among overweight/obese individuals is still controversial. The contradictory results could be due to use of estimated vitamin B12 intake and higher heterogeneity in studies regarding α- and β-diversity, along with the varied nature of the sample and methods in identifying microbial composition.

Interestingly, we found a significant correlation between dietary vitamin B12 intake and gut microbiota relative to BMI at the species and phylum levels. In the obese group, we found a significant negative correlation between *Akkermansia muciniphila* species and dietary vitamin B12 intake, and the significance was extended to the phylum level of Verrucomicrobia. The observed negative correlation between dietary vitamin B12 intake and *Akkermansia muciniphila* species is contrary to the findings of Gurwara et al. (2019), who showed a positive correlation between the abundance of short-chain fatty acid-producing *Akkermansia* and a vitamin B12-rich diet, thus providing positive effects [38]. The discrepancies may be attributed to the obese participants in our sample. Obesity is considered one of the metabolic conditions that have negative correlations with the *Akkermansia muciniphila* species [45]. Additionally, in the current study, our results revealed that, among total participants, *Bifidobacterium kashiwanohense* species (Actinobacteria phylum) and *Blautia wexlerae* (Firmicutes phylum) species were negatively associated with dietary vitamin B12 intake. This finding suggests that if dietary vitamin B12 intake is high, the abundance of *Blautia wexlerae* and *Bifidobacterium kashiwanohense* species may be reduced. Previous studies have demonstrated that vitamin B12 supplementation for vitamin B12-deficient patients significantly increased the abundance of Firmicutes and decreased Bacteroidetes [37].

A recent review investigated the association between vitamin B12 and gut microbiota with obesity, and reported that obesity-related gut microbiota dysbiosis may contribute to the altered vitamin host status [21]. According to the review, the status of vitamin B12 was related to various gut-related outcomes, such as alpha and beta diversity, relative abundance, and the production of short-chain fatty acids, which are the essential metabolites produced by the gut bacteria. These associations may be explained by three mechanisms: First, through gut microbiota, which are dependent on vitamin B12 and its analog “corrinoids” for methionine and nucleotide biosynthesis [46]. Approximately 2% of the intestinal corrinoids in humans are made by vitamin B12 [47]. Degnan and colleagues (2014) suggested that dietary corrinoids potentially influence the gut microbial system, inhibiting the growth of pathogenic bacteria while nourishing beneficial strains [48]. The second mechanism is that vitamin B12 plays a role in protecting the intestinal epithelium in numerous models of gastrointestinal diseases [34]. Finally, vitamin B12 may induce the fermentation and production of short-chain fatty acids in gut microbiota [35,38]. 

### Strengths and Limitations 

To the best of our knowledge, this is the first study to demonstrate a relationship between vitamin B12 levels and gut microbiota among young obese females. This study used the shotgun technique to analyze the α- and β-diversity of gut microbiota, which is considered a unique technique for assessing composition and diversity [29]. Additionally, our study assesses both vitamin B12 intake and serum levels. Nevertheless, our study had some limitations. The case–control study design cannot establish causation. Since all questionnaires used in this study relied on subjective reporting, there is a significant probability of recall bias during data collection. Furthermore, the study results cannot be generalized to the general Saudi population since it was performed only on females at a young age.

## 5. Conclusions

This study highlights the association between α- and β-diversity in gut microbiota and vitamin B12 levels among young females with different obesity levels. Our findings suggest that both α-diversity, which indicates the richness of the gut microbiome, and β-diversity, which measures the differences between communities, were inversely associated with low serum vitamin B12 in the obese sample. Obesity prevention and management is important worldwide, particularly in Saudi Arabia, where prevalence is increasing. Since both gut microbiota and low vitamin B12 levels play a role in the progression of obesity [49], sufficient vitamin B12 intake from diet or supplements may have positive impact on gut microbiota and obesity management, emphasizing the potential significance of this study for human health, especially among females. Further studies could provide insight into the associations between vitamin B12, obesity, and gut microbiota. 

## Figures and Tables

**Table 1:** Demographic characteristics and other data by serum vitamin B12 levels among normal-weight and obese participants, n = 92.

Variables	Normal			Obese		
	High Serum Vitamin B12 (>404.0 pg/mL)	Low Serum Vitamin B12 (≤404.0 pg/mL)	*p*-Value	High Serum Vitamin B12 (>404.0 pg/mL)	Low Serum Vitamin B12 (≤404.0 pg/mL)	*p*-Value
**Anthropometric Measurements**						
BMI (kg/m^2^)	21.8 ± 1.9	22.1 ± 1.9	0.68	35.9 ± 4.2	36.6 ± 5.2	0.68
WHR (ratio)	0.7 ± 0.0	0.7 ± 0.1	0.32	0.8 ± 0.1	0.8 ± 0.1	0.74
Body Fat (%)	35.2 ± 5.5	34.8 ± 5.6	0.83	51.7 ± 2.4	51.1 ± 3.5	0.56
Muscle mass (%)	31.8 ± 6.7	25.4 ± 11.8	**0.03**	26.2 ± 1.4	26.6 ± 2.1	0.51
**Blood Analysis**						
Total cholesterol (mmol/L)	3.4 ± 1.7	4.0 ± 1.7	0.29	3.9 ± 1.0	4.8 ± 1.0	**0.02**
HDL-cholesterol (mmol/L)	0.9 ± 0.4	1.1 ± 0.4	0.19	1.0 ± 0.2	1.0 ± 0.3	0.82
LDL-cholesterol (mmol/L)	2.5 ± 1.5	2.8 ± 1.5	0.53	2.8 ± 0.7	3.6 ± 1.0	**0.02**
Total cholesterol/HDL ratio	3.8 ± 1.4	3.8 ± 1.9	0.88	3.9 ± 0.5	5.0 ± 1.9	0.07
Triglyceride (mmol/L) #	0.5 (0.4–0.6)	0.7 (0.4–0.8)	0.18	0.8 (0.6–1.0)	1.0 (0.9–1.2)	**0.03**
FBG (mmol/L)	4.5 ± 0.9	4.5 ± 0.5	0.79	4.8 ± 0.8	4.8 ± 0.4	0.92
Insulin (µIU/mL) #	6.3 (5.0–9.3)	7.8 (5.1–9.5)	0.72	14.3 (12.5–19.5)	16.1 (12.2–20.5)	0.31
HOMA-IR #	1.2 (0.9–1.5)	1.4 (0.9–1.8)	0.59	3.4 (2.6–4.2)	3.7 (2.5–4.4)	0.57
HOMA-β #	112.0 (66.8–145.1)	127.1 (80.1–154.5)	0.36	210.0 (168.6–224.5)	252.8 (183.1–322.2)	0.36
Vitamin B12 (pg/mL)	620.4 (474.3–688.7)	334.7 (295.0–362.7)	**<0.001**	632.8 (427.7–781.6)	305.6 (236.9–354.6)	**<0.001**
**Dietary Intake**						
Energy intake (kcal/day)	3534.5 (3056.1–4454.9)	4236.5 (3351.1–5881.4)	0.29	3079.5 (1966.6–4611.7)	3041.0 (2637.3–4252.6)	0.95
Fat intake (%)	36.8 (33.6–42.1)	40.2 (34.8–45.7)	0.34	35.1 (25.6–41.2)	42.0 (34.0–47.2)	**0.03**
Vitamin B12 (mg/day)	8.9 (6.4–12.4)	8.9 (5.1–17.4)	0.94	6.9 (4.1–15.5)	6.9 (4.7–12.3)	0.90
Dairy (gm/1000 kcal)	81.5 (53.1–132.1)	75.7 (39.8–93.9)	0.29	115.3 (32.7–201.2)	99.3 (57.0–129.8)	0.91
Fruit (gm/1000 kcal)	124.3 (72.4–197.2)	98.9 (67.6–202.0)	0.56	164.0 (45.9–231.8)	70.2 (47.1–127.3)	0.18
Vegetables (gm/1000 kcal)	194.9 (134.0–220.6)	286.0 (177.4–310.5)	0.05	132.7 (89.7–271.1)	164.3 (124.6–241.3)	0.51
Egg (gm/day)	34.9 (10.0–72.8)	24.4 (12.0–42.5)	0.43	25.4 (4.0–68.2)	51.6 (8.4–94.3)	0.25
Chicken (gm/day)	101.4 (56.7–132.8)	64.3 (33.7–119.8)	0.50	70.1 (39.1–114.0)	50.0 (10.8–104.8)	0.15
Red meat (gm/day)	51.7 (34.2–72.7)	64.3 (40.9–96.4)	0.27	26.0 (18.5–37.2)	29.6 (6.8–52.9)	0.53

Note: Data presented as mean ± SD for normal variables whereas median (1st quartile–3rd quartile) for non-normal variables; n (%) for categorical variables; # indicates non-normal variables; *p* < 0.05 considered significant. Body mass index (BMI), waist-to-hip ratio (WHR), high-density lipoprotein (HDL), low-density lipoprotein (LDL), fasting blood glucose (FBG), homeostatic model assessment of insulin resistance (HOMA-IR), homeostatic model assessment of β-cell function (HOMA-β), gram (gm), milligram (mg).

**Table 2:** Gut microbiota composition by serum vitamin B12 levels among normal-weight and obese participants, n = 92.

Variables	Normal				Obese	
	High Serum Vitamin B12 (>404.0 pg/mL)	Low Serum Vitamin B12 (<404.0 pg/mL)	*p*-Value	High Serum Vitamin B12 (>404.0 pg/mL)	Low Serum Vitamin B12 (<404.0 pg/mL)	*p*-Value
**Firmicutes**	0.21 (0.17–0.30)	0.20 (0.16–0.22)	0.59	0.21 (0.16–0.27)	0.20 (0.13–0.30)	0.70
*Blautia wexlerae*	0.01 (0.002–0.01)	0.01 (0.002–0.01)	0.38	0.01 (0.003–0.01)	0.01 (0.004–0.01)	0.78
*Flavonifractor plautii*	0.001 (0.0003–0.001)	0.001 (0.001–0.001)	0.59	0.001 (0.0003–0.001)	0.001 (0.0004–0.002)	0.43
*Clostridium bolteae*	0.001 (0.0000–0.0006)	0.0004 (0.00–0.001)	0.80	0.00 (0.00–0.0008)	0.0002 (0.00–0.001)	0.82
*Faecalibacterium prausnitzii*	0.02 (0.01–0.03)	0.02 (0.01–0.03)	0.63	0.018 (0.02–0.03)	0.016 (0.01–0.03)	0.19
*Lactobacillus acidophilus*	0.00 (0.00–0.00)	0.00 (0.00–0.00)	0.71	0.00 (0.00–0.00)	0.00 (0.00–0.00)	0.73
*Clostridioides difficile*	0.00 (0.00–0.00)	0.00 (0.00–0.00)	0.40	0.00 (0.00–0.00)	0.00 (0.00–0.00)	0.95
**Bacteroidetes**	0.73 (0.55–0.79)	0.74 (0.69–0.77)	0.95	0.75 (0.66–0.81)	0.74 (0.67–0.83)	0.78
*Bacteroides* “unspecified species”	0.001 (0.0003–0.003)	0.002 (0.0004–0.005)	0.31	0.004 (0.001–0.004)	0.002 (0.0003–0.01)	0.57
*Bacteroides faecichinchillae*	0.00 (0.00–0.00)	0.00 (0.00–0.00)	0.91	0.00 (0.00–0.00)	0.00 (0.00–0.00)	0.73
*Bacteroides thetaiotaomicron*	0.01 (0.004–0.01)	0.01 (0.003–0.02)	0.74	0.01 (0.004–0.01)	0.01 (0.003–0.01)	0.82
*Bacteroides uniformis*	0.07 (0.03–0.09)	0.08 (0.05–0.08)	0.59	0.08 (0.04–0.13)	0.07 (0.04–0.09)	0.78
**Actinobacteria**	0.03 (0.02–0.06)	0.05 (0.03–0.07)	0.28	0.02 (0.003–0.05)	0.02 (0.02–0.04)	0.87
*Bifidobacterium adolescentis*	0.005 (0.00–0.02)	0.001 (0.00–0.02)	0.56	0.003 (0.0003–0.01)	0.001 (0.00–0.01)	0.70
*Bifidobacterium kashiwanohense*	0.00 (0.00–0.0003)	0.00 (0.00–0.003)	0.47	0.00 (0.00–0.001)	0.00 (0.00–0.0004)	0.95
*Bifidobacterium longum*	0.007 (0.002–0.01)	0.01 (0.001–0.01)	0.97	0.003 (0.00–0.009)	0.01 (0.001–0.01)	0.63
*Bifidobacterium merycicum*	0.00 (0.00–0.00)	0.00 (0.00–0.00)	0.44	0.00 (0.00–0.00)	0.00 (0.00–0.00)	0.82
*Bifidobacterium pseudocatenulatum*	0.0002 (0.00–0.002)	0.001 (0.00–0.005)	0.22	0.0003 (0.00–0.001)	0.0003 (0.00–0.003)	0.66
**Verrucomicrobia**	0.001 (0.00–0.0069)	0.0002 (0.00–0.001)	0.16	0.001 (0.00–0.01)	0.0002 (0.00–0.001)	0.80
*Akkermansia muciniphila*	0.002 (0.00–0.008)	0.0002 (0.00–0.001)	0.12	0.001 (0.00–0.01)	0.0002 (0.00–0.001)	0.51
**Proteobacteria**	0.02 (0.01–0.02)	0.01 (0.01–0.02)	0.23	0.01 (0.01–0.02)	0.10 (0.01–0.02)	0.43
**Synergistetes**	0.00 (0.00–0.00)	0.00 (0.00–0.00)	0.99	0.00 (0.00–0.00)	0.00 (0.00–0.00)	0.68
**Fusobacteria**	0.00 (0.00–0.00)	0.00 (0.00–0.00)	1.00	0.00 (0.00–0.00)	0.00 (0.00–0.00)	0.73
**Bacteria** “unspecified phylum”	0.00 (0.00–0.0004)	0.0002 (0.00–0.001)	0.51	0.0004 (0.00–0.001)	0.0002 (0.00–0.001)	0.75
**F/B** (ratio)	0.28 (0.21–0.57)	0.27 (0.20–0.31)	0.67	0.27 (0.20–0.41)	0.25 (0.16–0.45)	0.73

Note: Data presented as mean ± SD for normal continuous variables, median (1st quartile–3rd quartile) for non-normal continuous variables; *p*-value <0.05 considered significant. Bacteria written in bold indicate phylum, while those in italic indicate species. Firmicutes/Bacteroidetes (F/B).

**Table 3:** Correlation coefficients between vitamin B12 intake and gut microbiota among total and obese participants.

Variables		Vitamin B12 Intake	R	*p*-Value *
**Gut Microbiota**	**Total participants**	Bifidobacterium kashiwanohense species	−0.23	0.03
		Blautia wexlerae	−0.24	0.02
	**Obese participants**	Akkermansia muciniphila species	−0.32	0.03
		Verrucomicrobia phylum	−0.30	0.04
		Bacteroides “unspecified species”	0.30	0.04

* *p*-value < 0.05 considered significant.

## Data Availability

The date are available from the corresponding author.

## Supplementary Materials

The following supporting information can be downloaded at: https://www.mdpi.com/article/10.3390/foods11244007/s1, Table S1: General Characteristics of Participants Stratified by Serum Vitamin B12 Level, n = 92.
