# Supplementary material for: Vitamin B12 Status and Gut Microbiota among Saudi Females with Obesity
Source: Foods. 2022 Dec 11;11(24):4007. doi: 10.3390/foods11244007 (PMC9778531; doi:10.3390/foods11244007)
Supplement: Supplementary file 1 [file foods-11-04007-s001.zip › foods-2064233-supplementary.pdf]

Supplementary Table S1. General Characteristics of Participants Stratified by Serum Vitamin B12 Level, n=92

Variables	Vitamin B12		
	**High serum vitamin B12 > 404.0 pg/ml**	**Low serum vitamin B12≤ 404.0 pg/ml**	**P-value**
**Anthropometric Measurements**			
BMI (kg/m^2)	25.6 ± 6.9	31.8 ± 8.2	**<0.001**
WHR (ratio)	0.7 ± 0.1	0.8 ± 0.1	**0.05**
Body Fat (%)	39.7 ± 8.9	45.6 ± 8.8	**0.003**
Muscle mass (%)	30.4 ± 6.3	26.2 ± 6.7	**0.006**
**Blood Analysis**			
Total cholesterol (mmol/l)	3.6 ± 1.6	4.6 ± 1.3	**0.003**
HDL- cholesterol (mmol/l)	0.9 ± 0.3	1.1 ± 0.3	0.096
LDL- cholesterol (mmol/l)	2.5 ± 1.3	3.3 ± 1.2	**0.008**
Total cholesterol/HDL ratio	3.9 ± 1.2	4.6 ± 2.0	0.06
Triglyceride (mmol/l) #	0.5 (0.4 - 0.8)	0.9 (0.7 - 1.1)	**<0.001**
FBG (mmol/l)	4.6 ± 0.9	4.7 ± 0.5	0.237
Insulin (μIU/mL) #	7.3 (5.5 - 13.7)	12.5 (8.6 - 18.3)	**0.007**
HOMA-IR #	1.3 (1.0 - 3.0)	2.5 (1.5 - 3.9)	**0.006**
HOMA-β #	142.4 (90.7 - 208.7)	211.9 (135.7 - 310.6)	**0.007**
Vitamin B12 (pg/ml)	632.2 (474.3 - 724.1)	310.7 (267.9 - 359.7)	**<0.001**
**Dietary History**			
Energy intake (kcal/day)	3495.1 (2790.1 - 4454.9)	3383.2 (2658.3 - 4499.0)	0.93
Fat intake (%)	36.6 (32.7 - 41.2)	42.0 (34.4 - 46.7)	0.04
Vitamin B12 (mg/day)	8.7 (6.2 - 13.2)	7.6 (4.9 - 13.1)	0.46
Dairy (gm/1000kcal)	84.5 (53.1 - 132.8)	92.7 (51.6 - 123.8)	0.96
Fruit (gm/1000kcal)	126.5 (72.4 - 211.5)	79.0 (50.7 - 156.9)	0.04
Vegetables (gm/1000kcal)	174.1 (104.9 - 220.6)	182.5 (137.8 - 283.7)	0.38
Egg (gm/day)	25.7 (10.0 - 68.2)	42.6 (9.8 - 74.6)	0.59
Chicken (gm/day)	87.7 (55.0 - 124.3)	56.0 (12.4 - 109.8)	0.02
Red meat (gm/day)	46.5 (31.3 - 71.4)	39.1 (12.3 - 68.9)	0.30

Note: Data presented as mean ± SD for normal variables whereas median (1st quartile – 3rd quartile) for non-normal variables; n (%) for categorical variables; # indicates non-normal variables. p<0.05 considered significant. Body mass index (BMI), waist-to-hip ratio (WHR), high-density lipoprotein (HDL), low-density lipoprotein (LDL), fasting blood glucose (FBG), homeostatic model assessment of insulin resistance (HOMA-IR), homeostatic model assessment of β-cell function (HOMA-β), Gram (gm), Milligram (mg).
